# Urine-based assays for inpatients with HIV-associated tuberculosis in rural South Africa

**DOI:** 10.4102/sajhivmed.v26i1.1705

**Published:** 2025-06-17

**Authors:** Mbulelo Mntonintshi, Bianca Sossen, Hloni Bookholane, Aimee Lifson, Lisa Africa, René Goliath, Nicola Wearne, Andy Parrish, Graeme Meintjes

**Affiliations:** 1 Department of Family Medicine and Rural Health, Faculty of Medicine and Health Sciences, Walter Sisulu University, Mthatha, South Africa; 2 Department of Medicine, Faculty of Health Sciences, University of Cape Town, Cape Town, South Africa; 3 Department of Medicine, Mitchells Plain Hospital, Cape Town, South Africa; 4 Wellcome Centre for Infectious Disease Research in Africa, Institute of Infectious Disease and Molecular Medicine, University of Cape Town, Cape Town, South Africa; 5 Division of Nephrology and Hypertension, Faculty of Health Sciences, University of Cape Town, Cape Town, South Africa; 6 Department of Internal Medicine, Frere and Cecilia Makiwane Hospitals, Walter Sisulu University Central Deanery, Mthatha, South Africa; 7 Blizard Institute, Faculty of Medicine and Dentistry, Queen Mary University of London, London, United Kingdom

**Keywords:** tuberculosis, HIV, urine xpert ultra, AlereLAM, diagnostic, inpatient, non-sputum-based, pragmatic

## Abstract

**Background:**

Accurate non-sputum-based tuberculosis (TB) diagnostics are urgently needed to improve diagnostic yield and patient outcomes.

**Objectives:**

To compare the diagnostic accuracy and diagnostic yield of Urine Xpert Ultra (Urine-XPU) and Urine Determine^TM^ TB Lipoarabinomannan (LAM) antigen test (AlereLAM) against both a microbiological and composite reference standard (MRS and CRS) in a rural, routine care setting in South Africa.

**Method:**

Adults (≥ 18 years) with HIV had sputum, urine and blood collected for comprehensive TB testing shortly after admission. Additionally, focused assessment with sonography for HIV-associated TB (FASH) was performed. The MRS was defined by Xpert Ultra or culture-based tests for *Mycobacterium tuberculosis*. The CRS incorporated these mycobacterial tests, FASH findings, and clinical response to empiric TB treatment. Follow-up was conducted at 3 months.

**Results:**

A total of 206 participants were enrolled, with a median age of 39 years and 63% were female. Using the MRS the sensitivity of AlereLAM was 45.2% (95% confidence interval [CI]: 31.2–60.1) and Urine-XPU, 59.5% (95%CI: 44.5–73.0); and the specificity of AlereLAM was 93.6% (95%CI: 88.2–96.6) and Urine-XPU 95.0% (95%CI: 90.0% – 97.6%). Urine-XPU and AlereLAM performed better than sputum Xpert Ultra (Sputum-XPU) in patients with more severe illness. Additionally, Urine-XPU showed potential for accurately detecting rifampicin resistance.

**Conclusion:**

Urine-XPU and AlereLAM demonstrated comparable diagnostic accuracy for TB in hospitalised adults with HIV. Integrating Urine-XPU alongside AlereLAM and Sputum-XPU may improve timely and accurate diagnosis of TB and rifampicin resistance. Further research is required to optimise the diagnosis-to-treatment pathway.

**What this study adds:** In this study of adult inpatients with HIV from a South African rural setting, we show the diagnostic utility of Urine-XPU in addition to the currently available Sputum-XPU and AlereLAM.

## Introduction

Tuberculosis (TB) is the leading cause of death, hospitalisation and inpatient mortality in patients with HIV.^1,2,3^ This is partly because of imperfect diagnostic tests for HIV-associated TB, particularly in rural settings where resources are scarce, as evidenced by the frequency of post-mortem diagnosis of TB in autopsy studies.^4,5^

In people with HIV (PWH) who are admitted to hospital, the WHO-recommended investigations for TB include a rapid molecular test (e.g. Xpert MTB/RIF Ultra; [Cepheid, Sunnyvale, CA, United States]) on sputum, certain extra-pulmonary samples, and the Determine^TM^ TB Lipoarabinomannan (LAM) Ag assay (AlereLAM; Abbott, Chicago, IL, United States) on urine.^6^ TB is frequently extra-pulmonary and sputum pauci-bacillary in advanced HIV disease. In these patients there is often difficulty in obtaining sputum and subsequently in making a diagnosis.^7^ In comparison, urine is easy to obtain and non-invasive. There is a need for improved non-sputum-based diagnostics for TB in this patient group.^8^

Xpert tests are rapid, low complexity, and laboratory-based. Xpert MTB/RIF Ultra has superior sensitivity to the prior cartridge version (Xpert MTB/RIF) in patients with sputum smear negative or extrapulmonary TB, but slightly decreased specificity in sputum.^9,10^

The Xpert platform can also be used to test urine, and urine Xpert Ultra (Urine-XPU; Cepheid, Sunnyvale, CA, United States) can also confirm rifampicin sensitivity. In a multinational cohort including unselected inpatients and symptomatic outpatients, the sensitivity of Urine-XPU was similar to AlereLAM, but it had higher specificity and positive predictive value (PPV).^11^ In an assessment of diagnostic yield for patients with extrapulmonary TB (EPTB) in Tanzania, Urine-XPU had a yield of 26% in those with definite EPTB, and was the only positive test in 12% of their EPTB patients.^12^ In an early study with the original Xpert assay, urine testing was able to diagnose more TB patients than sputum within their first day of hospital admission.^13^

AlereLAM is a point-of-care TB test, with results available within 30 min. While it has relatively high specificity, its low sensitivity and inability to assess for rifampicin resistance limit its utility. Urine mycobacterial smear and culture are not recommended for the diagnosis of TB in PWH because of the low diagnostic yield.^14^

Despite evidence of test accuracy, diagnostic yield and improved patient outcomes, there is sparse research on real-world, pragmatic use of new TB tests (e.g. AlereLAM and Urine-XPU) and their effects on the TB diagnostic and treatment cascade.^15^ There is a need to investigate new TB tests in real-world healthcare settings, considering certain key aspects, including how the tests function within existing resources, infrastructure, and workflows, while also assessing their accuracy, reliability, and ability to inform treatment decisions.

In a resource-limited rural setting, there may be unique considerations with regard to the utility and feasibility of various TB tests in PWH who are hospitalised. Furthermore, there are limited available data in general regarding the diagnostic accuracy of Urine-XPU. The aim of the study was to assess the diagnostic accuracy, yield, and real-world feasibility of urine assays for TB diagnosis and treatment initiation in PWH who are admitted to hospital.

## Methods

### Study design and participants

We conducted a prospective observational cohort study, by enrolling consenting adult (≥ 18 years) PWH being admitted to the medical wards, regardless of symptoms or CD4 count, in Butterworth Hospital, rural Eastern Cape province, South Africa. Patients were not eligible if they were already on TB treatment within the last month. Participants were systematically investigated for TB using urine, sputum, and blood tests, as well as ultrasound with the focused assessment with sonography for HIV-associated tuberculosis (FASH) protocol.^16^ FASH ultrasounds were all performed by the same, single reader (MM) after training, and included assessment for the presence of: pericardial effusion, pleural effusion, ascites, focal liver or splenic lesions and/or intra-abdominal lymphadenopathy. Results of clinically-indicated, additional tests for TB that were performed by the treating clinicians were also captured into the study database. Participants were followed up 3 months later. At this 3-month visit, which was either conducted in person or telephonically, vital status was documented as well as whether the participant had been started on TB treatment and whether there had been a symptomatic response to this treatment.

### Ethical considerations

Ethical approval was obtained from Walter Sisulu University, Health Sciences Research Ethics Committee (015/2019) and the University of Cape Town Faculty of Health Sciences Human Research Ethics Committee (749/2018). Written informed consent was obtained from all the participants in the study. Study enrolment did not affect standard of care, and results were shared with treating clinicians once available.

### Procedures

Procedures were designed to reflect real-world clinical conditions. A trained research nurse looked for patients in the emergency department and in adult medical wards daily on weekdays in an attempt to identify all potentially eligible admitted patients consecutively. Patients were screened for eligibility if they self-reported living with HIV or if they agreed to voluntary counselling and testing and were found to have two rapid tests positive for HIV. If there were more patients identified than could be enrolled on a single day, a randomised selection procedure using an online tool (the List Randomizer – random.org) was followed.

Enrolled participants were asked about their medical history, medications and current symptoms using a standardised questionnaire. All study samples (urine, sputum and blood) were collected within 1–2 days of hospital admission, although not necessarily on the same day. Sputum induction was possible, when spontaneous sputum samples were not obtained, if participants agreed to this, and with oversight by the research nurse.

### Reference standards

The objectives of this study were to compare the diagnostic accuracy and diagnostic yield of Urine-XPU and AlereLAM (index tests) against both a microbiological and composite reference standard (MRS and CRS).

We defined MRS-positive participants as those with any study or clinically indicated test positive for *Mycobacterium tuberculosis* (MTB) on Xpert Ultra or mycobacterial culture. We defined MRS-negative participants as those with no tests positive for MTB. We defined CRS-positive participants to include those who were MRS positive, or those who were started on TB treatment by the routine treating clinicians, or who had a FASH-ultrasound that was suggestive of TB. CRS-negative participants were those with no test positive for MTB and who were not started on TB treatment by the routine treating clinicians.

Baseline reference standard study testing aimed to test all participants with urine mycobacterial culture, 1–2 sputum Xpert Ultra (Sputum-XPU), 1–2 sputum mycobacterial cultures, and a blood mycobacterial culture. For study sputum samples, each sputum was separated for Xpert Ultra and culture within the lab. All samples were processed at either an on-site or off-site accredited laboratory, following standardised protocols. All tests towards the reference standard were done in the accredited National Health Laboratory Service (NHLS), the largest diagnostic pathology service in South Africa. NHLS is responsible for supporting the national and provincial health departments in the delivery of healthcare. TB tests via the NHLS for the study hospital are performed offsite in Mthatha (approximately 120 km away).

Cultures were performed in mycobacteria growth indicator tubes (MGIT; Becton Dickinson, Franklin Lakes, NJ, United States) with liquid medium. Identification of MTB in cultures was conducted with antigen detection (MPT64 antigen detection and/or MTBDRplus, MTBC and CM/AS line probe assays [Hain Lifescience, Nehren, Germany]). Blood cultures were done in BACTEC^TM^ Myco/F Lytic culture vials (Becton Dickinson, Franklin Lakes, NJ, United States). Assessors of the reference standard were blinded to the index test results.

We also attempted to capture radiological evidence for TB, by systematically reviewing all chest X-rays that were requested by the routine treating clinicians. These results did not specifically inform the reference standards but results were available to treating clinicians, and may have been used as evidence to support TB treatment initiation and thereby could have affected CRS classification.

### Index testing

Index tests were AlereLAM and Urine-XPU. AlereLAM testing was done according to the manufacturer’s instructions on the same day as urine collection. Briefly, 60 µL urine was applied to the sample pad. After 25 min, test strips were read using the test’s reference scale card to confirm the grade-1 threshold for positivity. AlereLAM testing was ideally done within 2 h of the urine sample being provided. Invalid AlereLAM results (i.e., an absent control line) were to be repeated once from the same urine sample.

Urine-XPU tests were conducted at the accredited Global Clinical and Viral Laboratory (KwaZulu Natal, South Africa), approximately 550 km from the study hospital). Specimens for Urine-XPU were transported on ice for testing within 24 h. The urine was centrifuged at 3000 g for 15 min, following which the supernatant was removed, and the pellet resuspended in 0.75 mL phosphate-buffered saline and 1.5 mL of the Xpert sample reagent buffer. Any detectable MTB DNA in the urine samples was deemed positive, including trace readings. Error results were repeated once with the same urine sample. Assessors of the index tests were blinded to the microbiological reference standard. Index test results did not contribute to the MRS. Results of both index tests were shared with the routine treating clinicians once available and thereby could have impacted TB treatment decisions in real time, and thereby the CRS classification.

### Statistical analysis

Sample size estimation was based on a prior study at a district hospital in Cape Town, where unselected PWH being admitted to hospital had a prevalence of 32.6% of newly diagnosed active TB.^17^ It was also based on another planned analysis to assess the prevalence of Urine-XPU positivity in patients with and without renal impairment in this cohort, which will be reported elsewhere. We aimed to recruit 240 participants, with the assumption that 80 would be microbiologically diagnosed with active TB. This would allow us to demonstrate a difference in Urine-XPU positivity among two groups: 70% in those with renal impairment versus 35% in those with normal renal function (alpha = 0.05 and power = 80%).

To investigate the diagnostic accuracy of the index tests, we calculated the point estimates and 95% confidence intervals (CIs) for the sensitivity, specificity, PPV, and negative predictive value (NPV) – against the MRS and then the CRS. Participants without a valid index test result and those in whom reference standard assessment was neither positive nor negative were deemed ‘unclassifiable’, and could not contribute to the results of sensitivity, specificity, PPV, or NPV.

We calculated diagnostic yield against the MRS – both overall and in pre-specified subgroups – and defined this as the proportion of a test (or tests) positive, divided by the total number of non-index TB tests being positive for MTB. The diagnostic yield analysis did not require a valid index or reference test result, so if, for example, a participant could not produce a sputum sample or did not have a valid AlereLAM result, they were still able to contribute to the analysis of diagnostic yield. Subgroup assessment included those with TB bloodstream infection (TB-BSI), that is, those who had MTB detected on mycobacterial blood culture, according to CD4 count, haemoglobin, and venous lactate strata. We also assessed the association between positive bedside tests (AlereLAM, FASH ultrasound) versus Sputum-XPU versus Urine-XPU and TB treatment initiation. Reporting is in accordance with the Standards for Reporting of Diagnostic Accuracy Study guidelines (Online Appendix 1, Table 1-A1). The analysis was conducted in R (version 4.4.0; R Foundation for Statistical Computing, Vienna, Austria). Study data were collected and managed using REDCap electronic data capture tools hosted at UCT.^18,19^

## Results

**FIGURE 1:**
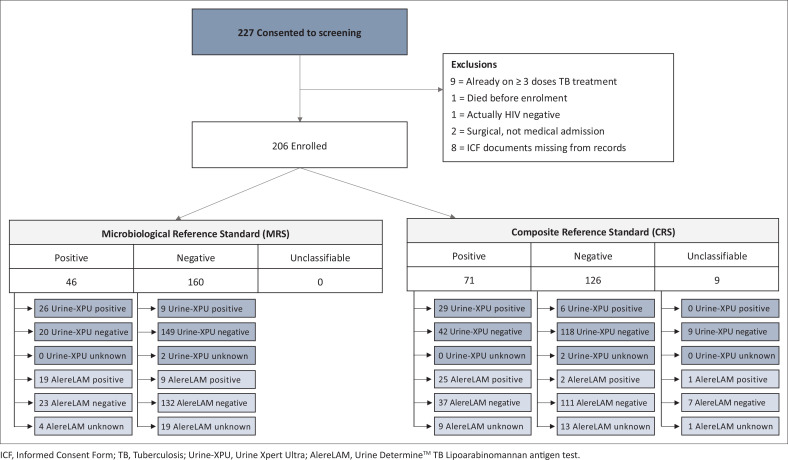
Participant flow diagram.

A total of 227 participants consented to be screened for this study, and 206 were eligible (Figure 1). Recruitment concluded slightly early, in March 2020, because of the Covid-19 pandemic.

The enrolled participants had a median age of 39 years and 62.9% (129/206) were female (Table 1). HIV was newly diagnosed in 35 (17.0%). MRS-positive participants were more likely to be in the antiretroviral therapy (ART)-interrupted group and had lower CD4 cell counts.

**TABLE 1:** Demographic, clinical characteristics and vital status outcomes of the cohort overall, and with stratification by the microbiological reference standard (MRS†).

Characteristic	Overall (*n* = 206)				MRS-positive† (*n* = 46)				MRS-negative† (*n* = 160)				*p*-value
	*n*	%	Median	IQR	*n*	%	Median	IQR	*n*	%	Median	IQR	
Age (years)	-	-	39	32–48	-	-	37	31–46	-	-	40	32–49	0.215
Female	129	62.6	-	-	24	52.2	-	-	105	65.6	-	-	0.097
**HIV history**	-	-	-	-	-	-	-	-	-	-	-	-	0.943
Newly diagnosed	35	17.0	-	-	8	17.4	-	-	27	16.9	-	-	-
Known with HIV prior	165	80.1	-	-	37	80.4	-	-	128	80.0	-	-	-
Unknown	6	2.9	-	-	1	2.2	-	-	5	3.1	-	-	-
**ART status**	-	-	-	-	-	-	-	-	-	-	-	-	0.051
On ART	135	65.5	-	-	25	54.3	-	-	110	68.8	-	-	-
ART naïve	28	13.6	-	-	5	10.9	-	-	23	14.4	-	-	-
Treatment interrupted	35	17.0	-	-	14	30.4	-	-	21	13.1	-	-	-
Status unknown	8	3.9	-	-	2	4.3	-	-	6	3.8	-	-	-
**CD4 count (cells/μL)**	-	-	166	45–320	-	-	63	27–239	-	-	175	54–362	0.027
≤ 200	79	38.3	-	-	21	45.7	-	-	58	36.3	-	-	-
> 200	57	27.7	-	-	9	19.6	-	-	48	30.0	-	-	-
Unknown CD4	70	34.0	-	-	16	34.8	-	-	54	33.8	-	-	-
Creatinine (μmol/L)	-	-	68	55–105	-	-	74	55–125	-	-	67	55–96	0.374
Haemoglobin (g/dL)	-	-	10.8	8.0–12.6	-	-	8.3	6.8–10.9	-	-	11.2	8.5–12.8	0.097
Venous lactate (mmol/L)	-	-	1.9	1.3–2.6	-	-	2.5	2.0–2.8	-	-	1.7	1.2–2.3	0.502
Prior TB history	74‡	36.8	-	-	16§	35.6	-	-	58¶	37.2	-	-	0.842
**TB treatment during study**	-	-	-	-	-	-	-	-	-	-	-	-	< 0.001
Treated	54	26.2	-	-	31	67.4	-	-	23	14.4	-	-	-
Not treated	140	68.0	-	-	12	26.1	-	-	128	80.0	-	-	-
Unknown	12	5.8	-	-	3	6.5	-	-	9	5.6	-	-	-
**Vital status at 10 weeks**	-	-	-	-	-	-	-	-	-	-	-	-	0.026
Alive	136	66.0	-	-	23	50.0	-	-	113	70.6	-	-	-
Died	58	28.2	-	-	20	43.5	-	-	38	23.8	-	-	-
LTFU	12	5.8	-	-	3	6.5	-	-	9	5.6	-	-	-

ART, antiretroviral therapy; IQR, Interquartile range; LTFU, lost to follow up; TB, Tuberculosis.

† , Microbiological reference standard (MRS)-positive participants were defined as those positive for *Mycobacterium tuberculosis* on Xpert Ultra or culture on any sputum or other sample, besides for the index tests, namely, Urine-XPU or AlereLAM. MRS-negative participants had no *Mycobacterium tuberculosis* detected on any of these reference tests;

‡ , 74 out of 201;

§ , 16 out of 45;

¶ , 58 out of 156.

Note: The *p*-values are for the comparison of that variable by MRS status, either with a c-squared test or Wilcoxon-rank sum test.

**FIGURE 2:**
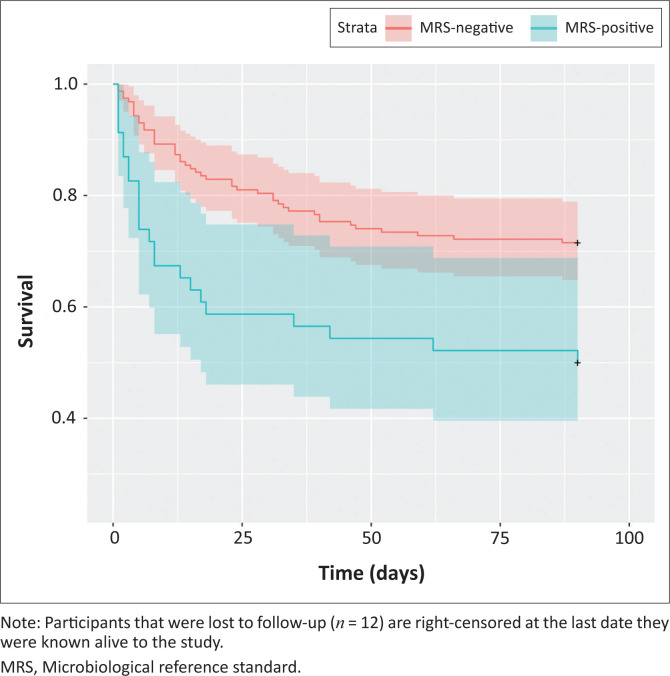
Survival curves in participants that were microbiological reference standard-positive versus negative.

Overall, approximately one in four participants in this cohort died within 90 days, with a higher percentage dying in the MRS-positive group (43.5%) compared to the MRS-negative group (23.8%), and MRS-positive participants died early (Figure 2). Twelve participants (5.8%) were lost to follow-up.

A total of 129/206 (62.6%) of the participants could produce at least one sputum sample, 31/206 (15.0%) of the cohort had a positive Sputum-XPU, and 18/206 (8.7%) had a positive sputum mycobacterial culture for MTB. Among those with positive Sputum-XPU results, four had trace-positive results. A total of 186/206 (90.3%) gave urine for mycobacterial culture, with 11/206 (5.3%) having a positive result for MTB, but 49/206 (23.8%) were contaminated. A mycobacterial blood culture was done in 194/206 (94.2%), and 22/206 (10.7%) of the cohort was positive for MTB on this test. Using any study or non-study test, excluding index tests, 46/206 (22.3%) were confirmed positive on the MRS.

In 16/129 (12.4%) of the participants, induction was known to be used for collection of any sputum sample. A further three participants were known to have required sputum induction, in order to obtain a second sputum sample after an initially spontaneously produced one. In 78/129 (60.5%) of those that produced a sputum sample, the participant was known to have done this spontaneously. In the remaining 35 participants, the sputum sampling method was unknown; for example, because of the sample having been collected by the routine treating team. In those known to have required sputum induction (*n* = 16), three (18.8%) were positive on Sputum-XPU and four (25%) were positive on Urine-XPU (with two participants therein being positive on both Sputum and Urine). In those known to have produced sputum spontaneously (*n* = 78), 20 (25.7%) were positive on Sputum-XPU and 12 (15.4%) were positive on Urine-XPU (with nine participants being positive on both Sputum and Urine).

A FASH ultrasound was done in 198/206 (96.1%), with 17/206 (8.3%) having features suggestive of abdominal, pleural, or pericardial TB. In 11/17 with a FASH suggestive of TB, MTB was also microbiologically confirmed.

With regard to the index tests, 204/206 (99.0%) had a Urine-XPU done and 35/206 (17.0%) of the cohort were positive by Urine-XPU, with 13/35 (37%) of these being trace positive for MTB. In comparison, 183/206 (88.8%) had a valid AlereLAM result, and 28/206 (13.6%) of the cohort was positive on AlereLAM.

### Diagnostic accuracy

All 206 participants were classifiable by the MRS, but 24 were excluded from diagnostic accuracy assessment because of either missing Urine-XPU or AlereLAM results. In 4/206 (1.9%), the AlereLAM result was indeterminate but not repeated, and in 19/206 (9.2%), AlereLAM was not done due to stock shortages on site. In 2/206, the Urine-XPU was not done because of repeat invalid results (on the same sample) (*n* = 1), and for an undocumented reason in one participant.

**FIGURE 3:**
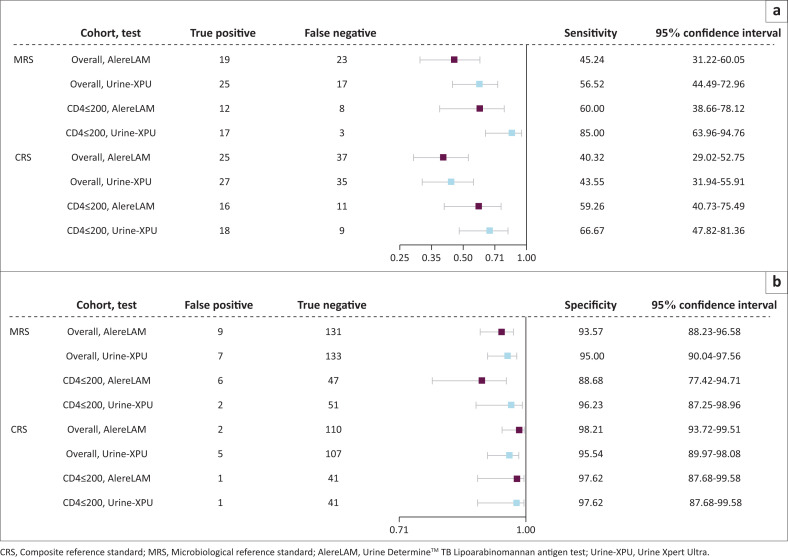
Diagnostic accuracy of AlereLAM and Urine-Xpert Ultra against the microbiological and composite reference standards, overall and in the subgroup with CD4 ≤ 200 cells/μL with sensitivity in (a) and specificity in (b).

Against the MRS overall, the sensitivity of AlereLAM was 45.2% (95%CI: 31.2 – 60.1) and Urine-XPU was 59.5% (95%CI: 44.5 – 73.0), and the specificity of AlereLAM was 93.6% (95%CI: 88.2 – 96.6), Urine-XPU was 95.0% (95%CI: 90.0 – 97.6%) (Figure 3). Against the MRS in those with a known CD4 ≤ 200 cells/uL, the sensitivity of AlereLAM was 60.0% (95%CI: 38.7 – 78.1) and Urine-XPU was 85.0% (95%CI: 64.0 – 94.8), and the specificity of AlereLAM was 88.7% (95%CI: 77.4 – 94.7) and for Urine-XPU was 96.2% (95%CI: 87.3% – 99.0%). Nine participants (4.4%) were unclassifiable by the CRS. Against the CRS overall, the sensitivity of AlereLAM was 40.3% (95%CI: 29.0 – 52.8) and Urine-XPU was 43.6% (95%CI: 31.9 – 55.9), and the specificity of AlereLAM was 98.2% (95%CI: 93.7 – 99.5) and Urine-XPU was 95.6% (95%CI: 90.0% – 98.1%). Clinical details of the five patients deemed false-positive on Urine-XPU by the CRS are in Online Appendix 1, Table 2-A1.

### Diagnostic yield

**FIGURE 4:**
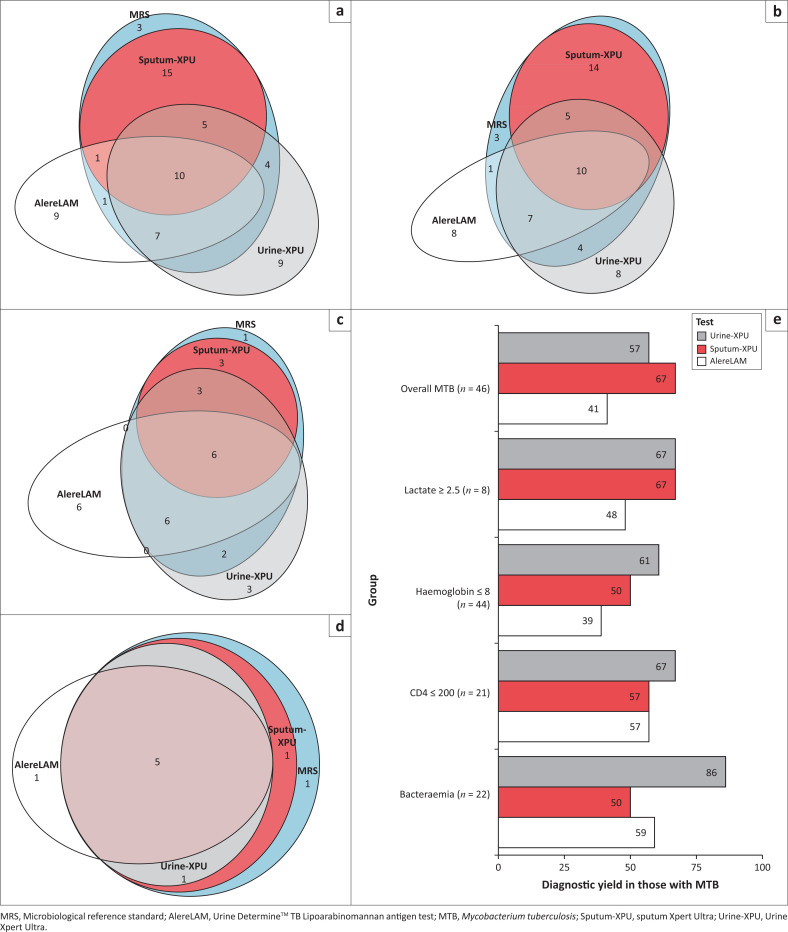
Euler plots of diagnostic yield of Urine-XPU (grey) and AlereLAM (white) in comparison to the microbiological reference standard (blue) and Sputum-XPU (red), in (a) the cohort overall, (b) those with haemoglobin ≤ 8 g/dL, (c) those with CD4 ≤ 200 cells/μL, and (d) those with venous lactate ≥ 2.5 mmol/L; (e) demonstrates the comparative diagnostic yield of Urine-XPU (grey), AlereLAM (white) and Sputum-XPU (red) in box plots of the cohort overall and various subgroups.

Of the 46 MRS-positive participants overall, 67.4% (*n* = 31) were positive by Sputum-XPU, 56.5% (*n* = 26) by Urine-XPU, and 41.3% (*n* = 19) by AlereLAM (Figure 4a). With all three rapid tests, 84.8% of participants with microbiologically confirmed TB were diagnosed. An additional nine participants were positive by Urine-XPU and nine other participants by AlereLAM, who were otherwise negative by all other MRS tests. The diagnostic yield of Urine-XPU was equal to or higher than that of Sputum-XPU in the subgroups with lactate ≥ 2.5 mmol/L, haemoglobin ≤ 8 g/dL, and CD4 ≤ 200 cells/µL and in those with TB-BSI (Figure 3b, c, d and e). The diagnostic yield of AlereLAM was equal to or higher than that of Sputum-XPU in the subgroups with CD4 ≤ 200 cells/µL, and in those with TB-BSI. Notably, the participant groups that were positive by AlereLAM versus Urine-XPU versus Sputum-XPU were incompletely overlapping. In a sensitivity analysis, if Urine-XPU positives were all presumed to be true positive TB cases, the diagnostic yield of Urine-XPU was 63.6% (*n* = 35/55), of Sputum-XPU was 56.4% (*n* = 31/55), and of AlereLAM was 34.5% (*n* = 19/55).

A total of 12 out of 22 participants (55%) with confirmed TB-BSI died within 90 days. In comparison, 41 out of 174 participants (24%) with negative blood cultures died within the same timeframe. Among the 22 participants with confirmed TB-BSI, Urine-XPU demonstrated the highest positivity rate (19 out of 22; 86%). AlereLAM was positive in 13 out of 22 of these participants (59%), while Sputum-XPU identified 11 out of 22 (50%). No participants in this cohort were diagnosed with disseminated non-tuberculous mycobacteria.

### Rifampicin susceptibility results

A total of 46/206 participants had TB tests positive for MTB, excluding that by Urine-XPU alone. In 42/46 (91.3%), rifampicin-sensitive MTB was detected, in 4/46 (8.7%), only an indeterminate rifampicin result was available across all TB tests (i.e., with results output = ‘MTB Trace’), and in none of these 42 participants was rifampicin-resistant MTB detected (Online Appendix 1, Table 3-A1). In three participants with rifampicin-sensitive MTB detected on their reference TB tests, the baseline Urine-XPU result was for rifampicin-resistant MTB (Online Appendix 1, Table 4-A1). In four participants with an unknown rifampicin susceptibility status from their reference TB tests, Urine-XPU was able to confirm their rifampicin susceptibility status.

### Treatment initiation

We noted that not all MRS-positive participants were initiated on treatment (Table 1) and therefore conducted a post-hoc analysis to assess whether there was an association between positive point-of-care, bedside tests, and treatment initiation, in comparison to those tests performed in an off-site laboratory, in this rural setting. In the 28 participants that had an AlereLAM positive result, 23 (82.1%) were initiated on treatment, while three were not and two had an unknown treatment status (reasons for lack of treatment initiation outlined in Online Appendix 1, Figure 1-A1: including 1/3 who died before treatment could be initiated). In the 17 participants who had a point-of-care FASH ultrasound suggestive of TB, 14 (82.4%) were initiated on TB treatment, while three were not (reasons in Online Appendix 1, Figure 1-A1: including 1/3 who died on day five of study). In comparison, Urine-XPU and Sputum-XPU are not performed at the point of care, and 23/35 (65.7%) of Urine-XPU-positive participants, as well as 22/31 (71.0%) of Sputum-XPU-positive participants, were initiated on TB treatment. In 5/11 Urine-XPU-positive and 3/7 Sputum-XPU-positive participants who were not initiated on TB treatment, these participants died, and this occurred between days 1 and 18 of the study (median 5 days), with these results having not yet been available or having been missed before the participant’s demise (Online Appendix 1, Figure 1-A1).

## Discussion

In a real-world study conducted in a rural setting of South Africa, Urine-XPU demonstrated good diagnostic accuracy and yield for adult inpatients with HIV. Both Urine-XPU and AlereLAM exhibited diagnostic performance that was equivalent or superior to Sputum-XPU in participants with features of more severe TB. We also found that point-of-care tests (e.g. AlereLAM or FASH) were associated with better connection of participants to TB treatment initiation, compared to tests performed in an off-site laboratory.

A high proportion of PWH being admitted to hospital have TB, and this cohort highlights the significant risk of early deterioration and death in this group, emphasising the need for rapid diagnosis and intervention. Notably, almost all participants were able to provide a urine specimen for testing, whereas only 63% were able to produce sputum. The use of urine-based tests may increase the likelihood of detecting TB in patients who would otherwise be missed by sputum-based tests.

Our study results were consistent with prior literature, in that Urine-XPU showed similar or improved diagnostic sensitivity and similar diagnostic specificity compared to AlereLAM.^11,20^ We also assessed diagnostic yield in clinically important patient subgroups, such as those with TB-BSI or features of TB sepsis, and this revealed that Urine-XPU performed equally to or better than Sputum-XPU or AlereLAM in these groups.

There have been previous reports of rifampicin susceptibility discrepancies between the older Xpert MTB/RIF cartridge on urine, compared to that detected from other TB tests.^21^ With the newer Xpert MTB/RIF Ultra cartridge on urine, these discrepancies were not seen in other studies.^11^ In our cohort, there were three participants with rifampicin resistance detected on their Urine-XPU, but rifampicin-sensitive MTB was detected on their other TB tests. In these three participants, it is still possible that this represents heteroresistance, but this might also represent false-positive rifampicin resistance results. Sequencing of these participants’ cultured isolates might have helped to further understand whether heteroresistance was possibly present, but this was not possible as part of this study.

Despite high pre-test probability of TB in this cohort, not all participants with TB detected had TB treatment initiated. Participants with positive point-of-care tests (AlereLAM and FASH ultrasound) more frequently had TB treatment started compared to those with positive laboratory-based tests (Urine-XPU and Sputum-XPU). However, a substantial number of participants with positive laboratory-based tests died before treatment could be initiated, highlighting the importance of urgent diagnosis and treatment initiation in this patient population. In other settings and countries, the disconnect between diagnosis and treatment has been well-described.^15,22,23^ Our results emphasise the benefits of point-of-care testing or near-patient testing in on-site laboratories, in improving TB diagnosis and rapid treatment initiation, as well as the need for further research to address the ongoing barriers to treatment initiation after a diagnosis is confirmed.

The strengths of this study include that it was carried out in a rural setting, with a high burden of TB and HIV, targeting the population that would benefit most from the study findings. Despite being in a rural setting with limited resources, our diagnostic accuracy assessment was conducted against an extensive MRS. We were also able to go beyond the diagnostic accuracy and yield assessments, and study the prognostic value of certain tests and their ability to connect participants with TB treatment initiation.

While our study provides valuable insights, there are important limitations to consider. Our study was conducted in a single hospital, and the results might not be reproducible elsewhere. Due to the pragmatic nature of our study, we also had missing data on important variables such as CD4 counts. In light of the index tests being communicated to the treating clinicians in real time, this could have impacted on the CRS classification and our interpretation thereof. Future research should include assessment in different settings, addressing barriers to TB treatment initiation and implementation science approaches to assess and support real-world feasibility and roll-out of new TB diagnostic tests.

AlereLAM can be performed at the point of care and was associated with higher rates of treatment initiation compared with Urine-XPU. Urine-XPU, however, demonstrated sensitivity gains and similar specificity, along with higher diagnostic yield especially in those participants with features of severe TB. With Urine-XPU available in addition to AlereLAM and Sputum-XPU, 85% of TB participants in this cohort were positive on a rapid TB diagnostic test. Urine-XPU should be integrated into routine clinical practice, alongside AlereLAM and Sputum-XPU, for PWH being admitted to hospital, especially for those who are unable to produce sputum.
